# Identification of Biomarkers for Predicting Lymph Node Metastasis of Stomach Cancer Using Clinical DNA Methylation Data

**DOI:** 10.1155/2017/5745724

**Published:** 2017-08-29

**Authors:** Jun Wu, Yawen Xiao, Chao Xia, Fan Yang, Hua Li, Zhifeng Shao, Zongli Lin, Xiaodong Zhao

**Affiliations:** ^1^School of Biomedical Engineering, Shanghai Jiao Tong University, Shanghai, China; ^2^Department of Automation, Shanghai Jiao Tong University, Shanghai, China; ^3^School of Communications and Electronics, Jiangxi Science & Technology Normal University, Nanchang, China; ^4^Charles L. Brown Department of Electrical and Computer Engineering, University of Virginia, Charlottesville, VA, USA

## Abstract

**Background:**

Lymph node (LN) metastasis was an independent risk factor for stomach cancer recurrence, and the presence of LN metastasis has great influence on the overall survival of stomach cancer patients. Thus, accurate prediction of the presence of lymph node metastasis can provide guarantee of credible prognosis evaluation of stomach cancer patients. Recently, increasing evidence demonstrated that the aberrant DNA methylation first appears before symptoms of the disease become clinically apparent.

**Objective:**

Selecting key biomarkers for LN metastasis presence prediction for stomach cancer using clinical DNA methylation based on a machine learning method.

**Methods:**

To reduce the overfitting risk of prediction task, we applied a three-step feature selection method according to the property of DNA methylation data.

**Results:**

The feature selection procedure extracted several cancer-related and lymph node metastasis-related genes, such as TP73, PDX1, FUT8, HOXD1, NMT1, and SEMA3E. The prediction performance was evaluated on the public DNA methylation dataset. The results showed that the three-step feature procedure can largely improve the prediction performance and implied the reliability of the biomarkers selected.

**Conclusions:**

With the selected biomarkers, the prediction method can achieve higher accuracy in detecting LN metastasis and the results also proved the reliability of the selected biomarkers indirectly.

## 1. Introduction

According to the recent reports of the World Health Organization (WHO), stomach cancer is the fifth most common cancer in the world and more than 70% of the new cases of stomach cancer occurred in developing countries (mainly in China) [1, 2]. The early stage of stomach cancer, which is defined as stomach cancer limited to the mucosa or submucosa and irrelevant to the presence or absence of lymph node (LN) metastasis, confers a survival rate of greater than 90% in 5 years in many centers [3]. However, even in the early stage, it was reported that the incidence of LN metastasis was 14.1% overall and was 4.8 to 23.6% depending on cancer depth [4, 5]. Many researchers demonstrated that LN metastasis is an independent risk factor for stomach cancer recurrence in patients following curative resection, and the overall survival of LN metastasis-negative stomach cancer patient is significantly longer than that of LN metastasis-positive patients [6, 7]. Therefore, it is certain that an accurate LN metastasis presence prediction can provide the guarantee of credible prognosis evaluation of stomach cancer patients.

Traditionally, LN metastasis diagnosis is mainly implemented by preoperative imaging such as abdominal ultrasonography (US) and computed tomography (CT), but their diagnostic accuracy is limited. It was reported that the detection rate of lymph nodes around the stomach was 18.7% in CT and 5.0% in US [8]. Endoscopic ultrasonography (EUS) is an effective approach and generally provides a more accurate prediction of the tumor stage than does CT. However, EUS-based prediction accuracy for LN is only slightly greater as compared to CT [4].

Recently, increasing evidences suggest the critical role of DNA methylation in human carcinogenesis [9, 10]. Aberrant DNA methylation is one of the common alterations in carcinogenesis, and it first appears before symptoms of the disease become clinically apparent [11–13]. In addition, aberrant DNA methylation can promote the progression of disease [14]. With the development of high-throughput technology, plenty of DNA methylation data are available for cancer prediction and biomarker identification [15–18]. Inspired by these applications, in this study, we used the DNA methylation data to categorize the incidence of LN metastasis in stomach cancer through a machine learning method. Considering the high-dimensionality and high-noisiness of the DNA methylation data, there are still several challenges to achieve the categorization. In contrast to the large number of features (probes), the small number of cancer samples available for training may lead to the degradation of classification performance and raise the risk of overfitting [19]. It is natural and perhaps essential to employ a feature selection step to obtain a feature set which only consists of genes contributing positively to the classification without redundant features. The key benefits of performing feature selection are reducing overfitting, improving accuracy, and reducing training time. Beyond that, feature selection in cancer research can help researchers to identify key carcinogenic markers and accurate prediction can provide references for clinical implementation. The feature selection methods mainly can be divided into three categories, which are the filter, wrapper, and embedded methods [20–23]. The filter methods use a measure to score feature subsets while the wrapper methods use a predictive model to score. With the wrapper method, different feature sets are generated and an optimal engine, such as genetic method [24], simulated annealing method [25], and particle swarm optimization method [26], is selected to search a set of features that best distinguish the training samples of different classes. Embedded methods are the catch-all group of techniques which perform feature selection as part of the model learning process.

In this study, we grouped the data of stomach cancer into three categories, normal, LN metastasis negative, and LN metastasis positive, according to the clinical information. A three-step feature selection method was applied to identify the key genes. To evaluate the reliability of the selected biomarkers, we introduced the random forest algorithm to predict the categories with and without the three-step feature selection method. The results showed that the prediction accuracy was largely improved with the selected biomarkers, and it also proved the reliability indirectly.

## 2. Results

### 2.1. Feature Selection

Feature selection is commonly used to remove the irrelevant and redundant features from the original feature set. The minimum redundancy maximum relevance (mRMR) feature selection method is a feature selection method for finding a set of features that have the highest relevance with the target class and are also maximally dissimilar to each other based on the mutual information theory. However, mRMR is computationally expensive. In our paper, the differential methylation analysis was integrated with mRMR to achieve the preliminary feature selection. To further obtain the most informative feature for classification, an embedded feature selection method with genetic algorithm was introduced to get the final optimal features.

#### 2.1.1. Feature Selection with Differential Methylation Region (DMR) Analysis

**Figure 1:**
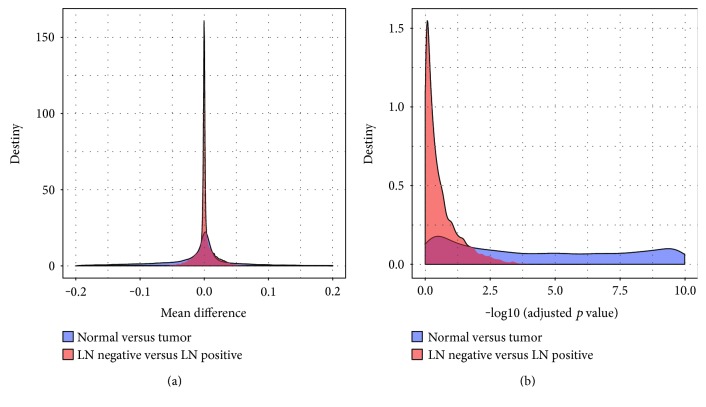
The density of the mean difference and BH-adjusted *p* value of the two comparisons. (a) The density of the mean difference of normal versus cancer comparison and LN negative versus LN positive comparison. (b) The density of the log10 BH-adjusted *p* value of normal versus cancer comparison and LN negative versus LN positive comparison.

To preliminarily obtain the probes that are closely related to the phenotype, DMR analysis, which aimed to identify significantly methylated probes between different phenotypes, was applied. We compared the methylation status of each probes in the normal samples within the cancer samples and the methylation status of probes in the LN-negative samples within the LN-positive samples. Differentially methylated probes were determined with the Mann–Whitney *U* test. The density of the mean difference and the Benjamin-Hochberg- (BH-) adjusted *p* value of the two comparisons were shown in Figure 1(), from which we can see that the methylation patterns were much more similar in the LN-negative and LN-positive samples than in the normal samples and cancer samples. The appearance indicated that the thresholds used for selecting significantly differentially methylated probes must be different according to the two comparisons. For the comparison of normal versus cancer, we selected probes with an adjusted *p* value less than 1*E*−5 and an absolute mean difference greater than 0.2 as significantly differentially methylated probes. For the comparison of LN negative versus LN positive, the threshold for the adjusted *p* value and absolute mean difference was set as 0.01 and 0.02, respectively. With such criteria, we identified 1077 and 275 as significantly differentially methylated probes in the two comparisons. There were only 33 probes shared by both.

#### 2.1.2. Feature Selection with the mRMR Method

**Figure 2:**
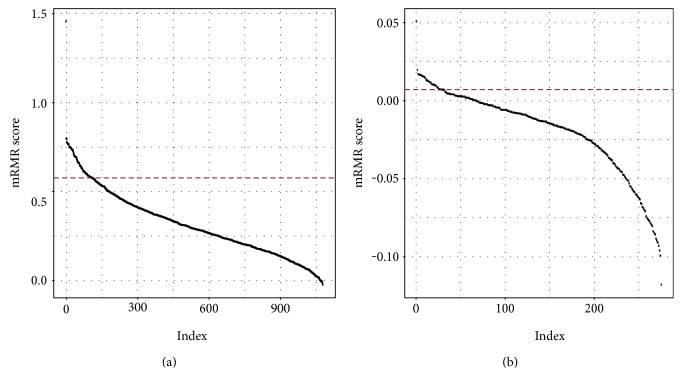
The distribution of mRMR scores with respect to features. The dashed line corresponds to the 10% cutoff used. (a) Normal versus cancer. (b) LN negative versus LN positive.

The classic mRMR method was applied to filter the probes selected previously, and the probes were ranked according to their score. Since there is no explicit threshold, only the top 10% probes were left and these probes were used as input to the next feature selection step. The results of mRMR filtering were shown in Figure 2, from which we can see that the scores in respect to the LN negative versus LN positive comparison were extremely low. The results implied that the LN-negative samples and LN-positive samples were very indistinct.

#### 2.1.3. Feature Selection with Genetic Algorithm

Performing feature selection with genetic algorithm requires conceptualizing the processing of feature selection as an optimization problem and encoded the solution as binary. In this paper, random forest algorithm was used as the fit function during the genetic algorithm and the receiver operating characteristic (ROC) was used to measure the fitness. The details will be discussed later in the section of Materials and Methods. The normal versus cancer classification and LN negative versus LN positive classification were treated independently.

**Figure 3:**
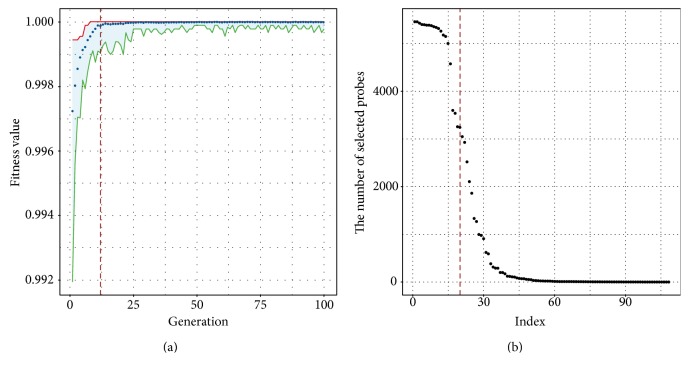
The results of genetic algorithm-based feature selection with respect to the normal versus tumor classification. (a) The fitness improvement in the process of iteration. (b) The distribution of the number of selected probes.

During the genetic algorithm in respect to the normal versus tumor classification, the ROC value summary in each iteration was shown in Figure 3(a), from which we can see that almost all the solutions can give a high fitness value. From this plot, we can see that after 12 iterations, the mean fitness hovered around 0.9999. We collected all the best solutions after each of the 12 iterations and simply summarized how many times a probe had been selected. The distribution of the number of selected probes were shown in Figure 3(b), and we selected the top 20 probes as the final features used for classification. According to the genomic locations, the 20 probes were associated to 39 genes including well-known cancer-related genes, such as *TP73*, *PDX1*, and *FUT8* [27–29].

**Figure 4:**
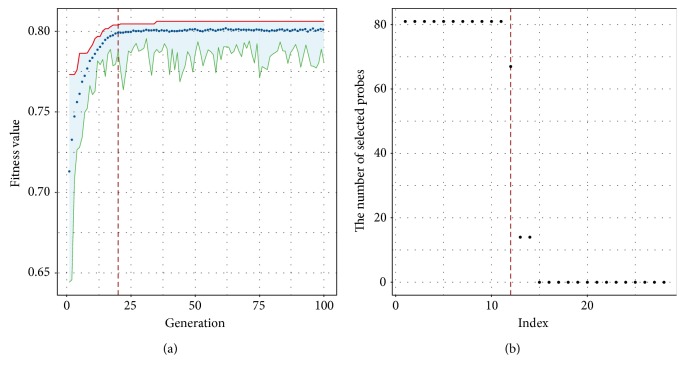
The results of genetic algorithm-based feature selection with respect to the LN negative versus LN positive classification. (b) The fitness improvement in the process of iteration. (a) The distribution of the number of selected probes.

The results of genetic algorithm in respect to the LN negative versus LN positive classification were shown in Figure 4(a), from which we can find that even after 100 iterations, the fitness is still not much greater than 0.8. This result also implied the indistinctness between the LN-negative and LN-positive samples. The mean fitness hovered around 0.8 after iteration 20. Similarly, we collected all the best solutions after each 20 iterations, and the distribution of the number of selected probes was shown in Figure 4(b). Finally, 12 probes were chosen for the final classification and associated with 14 genes including several lymph node metastasis-related genes, such as *HOXD1*, *NMT1*, and *SEMA3E* [30].

### 2.2. Classification Performance Evaluation

**Figure 5:**
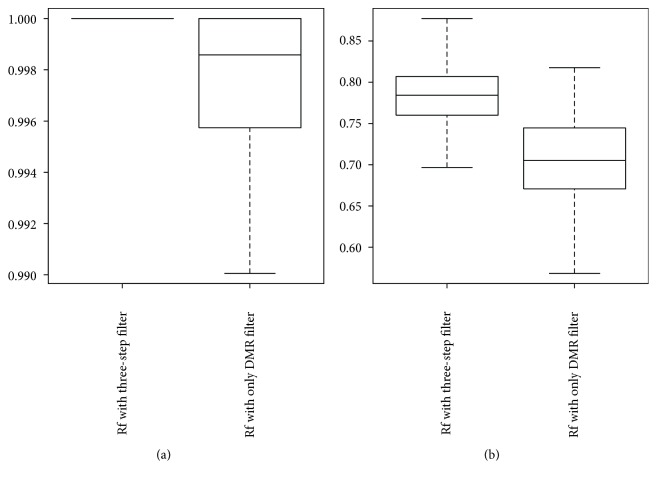
The distribution of the AUC value with different methods. (a) AUC value with different methods with respect to the normal versus tumor classification. (b) AUC value with different methods with respect to the LN negative versus LN positive classification.

To illustrate the necessity and effectiveness of the feature selection procedure, we compared the performance of the random forest using the three-step-selected probes with the random forest using only the differentially methylated probes. We randomly generated 100 training and testing data for evaluation, and the AUROC (area under ROC curve) value was used as measurement. The AUROC value of a classifier described the probability that the classifier will rank a randomly chosen positive instance higher than a randomly chosen negative instance. Simply put that a larger value of the AUROC means a higher discriminatory power. The box plots in Figure 5 shown below were the distribution of the AUC values of the prediction in respect to the normal versus tumor and LN negative versus LN positive.

From the plots, we can see that with the three-step feature selection procedure, the classifier can give a better performance in respect to both the normal versus tumor and LN negative versus LN positive classifications compared to with only the DMR analysis. Moreover, we also can find that the three-step feature selection or DMR only analysis gives good performance (AUC value all greater than 0.99) for the normal versus tumor classification.

## 3. Materials and Methods

### 3.1. DNA Methylation Dataset and DMR Analysis

The clinical data and the TCGA level 3 DNA methylation data were downloaded from The Cancer Genome Atlas (TCGA) project [31]. Only the samples with clear clinical diagnosis were used in the study. The details were shown in Table 1.

To identify differentially methylated probes, for each probe, we ranked the samples and compared only the lower methylation quintile sample to the upper methylation quintile sample between two phenotypes using the Mann–Whitney *U* test. The BH-adjusted *p* value and mean methylation difference were used to guide the identification.

### 3.2. Genetic Algorithm

Genetic algorithms are optimization tools that search the solution through simulating the evolution of random variation and natural selection. For feature selection, the individuals are subsets of candidate features that are encoded as binary and the value indicated that a feature is either included or not in the subset. The parameters used for the genetic algorithm were set as follow [19]:

- Population size: 100
- Maximum number of generations: 100
- Selection method: tournament selection with size = 2
- Elitism rate: 10 individuals
- Crossover: 2-point crossover with probability 0.6
- Mutation: random mutation with probability 0.05

The initial population was created by producing chromosomes with a random 30% of the predictors. The fitness function of every individual was defined as the ROC value of the classification method.

## 4. Conclusions

Stomach cancer is the fifth most common cancer in the world, and most of the new cases occurred in developing countries, especially in China. Recently, more and more evidence demonstrated that LN metastasis was an independent risk factor for stomach cancer recurrence in patients following curative resection, and the overall survival of LN metastasis-negative stomach cancer patients is significantly longer than that of LN metastasis-positive patients.

Based on the critical role of DNA methylation in human carcinogenesis, in this study, we focused on the prediction of the LN metastasis status using the DNA methylation data. However, considering the inherent disadvantage of DNA methylation data, such as the limited sample number compared to the large number of probes, we applied a three-step feature selection procedure to extract a small subset of representative features. First, we applied the differential methylation analysis to identify the significantly methylated probes between different phenotypes. Then, an mRMR method was introduced to remove the redundant feature obtained in the first filter step. Finally, a wrapper method based on genetic algorithm was used to achieve the final feature selection. We obtained 20 probes related to 39 genes which were inputs of the prediction in respect to normal versus tumor, and 12 probes related to 14 genes were input to the prediction in respect to LN negative versus LN positive (see Table 2). These genes related to the selected probes are mostly associated with cancer and LN metastasis, such as *TP73*, *PDX1*, *FUT8*, *HOXD1*, *NMT1*, and *SEMA3E*.

To evaluate the effect of three-step feature selection to the prediction performance, we downloaded the DNA methylation data and clinical data from the TCGA project. The AUROC value was used as the performance measurement. The experiment results showed that the three-step feature selection can largely improve the performance of prediction, especially predicting LN negative versus LN positive. The source code used in this paper can be obtained at https://git.oschina.net/junwu302/codes/m2gonkax18sfhdvl3e0b932.

## Figures and Tables

**Table 1:** The sample number for each phenotype.

Normal	Cancer		
	LN negative	LN positive	Unclassified
27	94	189	12

**Table 2:** Identified biomarkers for each prediction.

Normal versus tumor biomarkers	LN negative versus LN positive biomarkers
*SLC39A5*, *C3orf32*, *TP73*, *CD1B*, *PCDHGA4*, *PCDHGA11*, *PCDHGA9*, *PCDHGA1*, *PCDHGB1*, *PCDHGB6*, *PCDHGA12*, *PCDHGB3*, *PCDHGB7*, *PCDHGA6*, *PCDHGA8*, *PCDHGA10*, *PCDHGA5*, *PCDHGB4*, *PCDHGA3*, *PCDHGA2*, *PCDHGB2*, *PCDHGA7*, *PCDHGB5*, *C20orf197*, *SLC16A5*, *FUT8*, *SLC15A2*, *C17orf93*, *PRAC*, *OCLN*, *TMEM144*, *FGF2*, *PDX1*, *CCL1*, *LILRB5*, *LCE3D*, *GPR45*, *LPO*, *CGB5*	*LAT2*, *TTC13*, *ARV1*, *NMT1*, *DCAKD*, *GJA1*, *OR7A17*, *LOX*, *KRT19*, *ZNF655*, *KRTAP4*-*4*, *TAAR5*, *SEMA3E*, *HOXD1*
